# Hairy Garlic (*Allium subhirsutum*) from Sicily (Italy): LC-DAD-MS^n^ Analysis of Secondary Metabolites and In Vitro Biological Properties

**DOI:** 10.3390/molecules25122837

**Published:** 2020-06-19

**Authors:** Stefania Sut, Filippo Maggi, Sara Bruno, Natale Badalamenti, Luana Quassinti, Massimo Bramucci, Daniela Beghelli, Giulio Lupidi, Stefano Dall’Acqua

**Affiliations:** 1 Department of Agronomy Food, Natural Resources, Animals and Environment (DAFNAE), University of Padova, 35020 Legnaro, Italy; stefania_sut@hotmail.it; 2 School of Pharmacy, University of Camerino, I-62032 Camerino, Italy; filippo.maggi@unicam.it (F.M.); luana.quassinti@unicam.it (L.Q.); massimo.bramucci@unicam.it (M.B.); giulio.lupidi@unicam.it (G.L.); 3 Department of Biological, Chemical and Pharmaceutical Sciences and Technologies (STEBICEF), University of Palermo, IT-90128 Palermo, Italy; sara.br1996@gmail.com (S.B.); natale92@live.it (N.B.); 4 School of Biosciences and Veterinary Medicine, University of Camerino, I-62032 Camerino, Italy; daniela.beghelli@unicam.it; 5 Department of Pharmaceutical and Pharmacological Sciences, University of Padova, 35128 Padova, Italy

**Keywords:** plant extracts, hairy garlic, ethanolic extracts, acetylcholinesterase inhibitor, antioxidant activity, cytotoxicity

## Abstract

*Allium subhirsutum*, known as hairy garlic, is a bulbous plant widespread in the Mediterranean area and locally used as a food and spice. In the present study, the chemical profile of the ethanolic extracts from bulbs (BE) and aerial parts (APE) were analyzed by HPLC-ESI-MS^n^, and antioxidant properties were evaluated by DPPH, ABTS and TEAC assays. The traditional use in the diet, and the well documented biological activity of *Allium* species suggest a potential as a new nutraceutical. For this reason, the potential usefulness of this food can be considered in the treatment and prevention of degenerative Alzheimer disease. For this reason, acetylcholinesterase inhibitory property was investigated. Furthermore, due to the observed presence of sulfur-containing and phenolic constituents, the cytotoxicity on tumor cells line was investigated. Results revealed significant AChE inhibitory activity for BE and APE. Both extracts exhibited also moderate antioxidant properties in the in vitro assays. Finally, limited cytotoxic activity was observed towards Human colon carcinoma and adenocarcinoma cell line, with differences between the individual parts tested. HPLC-ESI-MS^n^ analysis showed that hairy garlic is a good source of sulphur compounds, flavonoids and phenylpropanoids derivatives, thus being a valid alternative to the common garlic (*A. sativum*). This work opens new opportunities for the application of *A. subhirsutum* as a health-promoting food.

## 1. Introduction

*Allium* is the largest genus of the Alliaceae family, formerly considered part of Liliaceae and Amaryllidaceae, and comprises more than 700 species distributed all over Europe, North America, Northern Africa and Asia, each differing in taste, form and color, but close in biochemical, phytochemicals and nutraceutical content [1]. Since ancient times, *Allium* species have been used in folklore of many cultures as food, spices or herbal remedies, and nowadays several members of this genus such as *A. cepa* L., *A. sativum* L., *A. ampeloprasum* L., *A. schoenoprasum* L., and *A. ascalonicum* L. are of high economic value not only for their use as a spice in traditional recipes and their taste but also for their action as bio-preservatives [2,3]. Species of the *Allium* genus are rich in flavonoids, saponins, sapogenins, and volatile sulphur compounds, and their characteristic organoleptic properties derive from the presence of non-volatile flavor precursors, alk(en)yl-L-cysteine sulfoxides. When the plant tissue is disrupted, the enzyme alliinase converts alk(en)yl-L-cysteine sulfoxides into the corresponding alk(en)yl thiosulfinates, which are transformed after cooking into a complex mixture of approximately 50 sulphur compounds, namely alk(en)yl(poly)sulphides [4]. Many sulphur compounds found in *Allium* species are responsible for several biological properties such as antimicrobial [5], antiatherosclerotic [6], antitumorigenetic [7], immunomodulatory [8] antiprotozoal, antioxidant, antihypertensive, hypolipidemic, hepatoprotective, and antithrombotic activities [4]. Moreover, other classes of constituents such as flavonoids, hydroxycinnamic acid derivatives and phenylpropanoid amide derivatives of tyramine or related compounds have been reported in *Allium* species [9,10,11].

*Allium subhirsutum* L., known as hairy garlic (known in Italy as “aglio pelosetto”), is a bulbous plant widespread in the Mediterranean Region from Spain and the Canary Island to Turkey and Palestine, Morocco, and Egypt. Within Europe, the plant has been reported from Spain, France, Italy, Greece, Turkey, Cyprus, and the former Yugoslavia. It is a perennial herb up to 50 cm tall. Leaves are long, up to 15 mm across, tapering toward the tip, with hairs along the margins (hence the name “hairy garlic”). The umbel contains only a few flowers, white with thin pink midveins [12].

The bulb, about 15 mm in diameter, is eaten raw or cooked. It is used like garlic as a flavoring in salads and cooked foods. The flavor is somewhat milder with a slight sweetness, and it can be used in much greater quantities than garlic. The leaves and flowers are also eaten raw or cooked and they have a pleasant texture, slightly sweet with a mild garlic flavor [13].

*A. subhirsutum* has been usually employed in the Italian popular medicine, especially in Sardinia where it is known as “sigajone”, for its anti-haemorrhoidal and purifying action. Furthermore, the whole plant is used to flavor salads, while the flowers and bulbs, cut into small pieces, are used as such or as a flavoring of several dishes, also to benefit of their properties on blood circulation due to the presence of alliin [14].

Many research articles have been published during the past years related to the possible health-promoting effects of garlic [8], focusing on immunomodulatory, anti-inflammatory, and antioxidant effects. The broad range of cardiovascular protection afforded by garlic extract may be extended to a protective effect on the brain, helping to reduce the risk of dementia, including AD [15]. In vitro and in vivo animal studies have demonstrated the mechanisms of action of garlic extract, which involve its antioxidant, anti-inflammatory and modulatory effects on neurotransmitter function in the brain regions that are associated with the pathogenesis of AD [16]. Thus, the evaluation of the inhibition of cholinesterase enzymes may provide a further basic contribution regarding the possible role of this food as a useful complementary agent for the management/prevention of neurodegenerative diseases.

Few papers have investigated this garlic species so far. Štajner et al., reported on the antioxidant properties of *A. subhirsutum* bulbs collected in Serbia [17], and quite recently the headspace volatiles of its crushed bulbs collected in Turkey were analyzed [18]. Recently, *A. nigrum* L. and *A. subhirsutum* were investigated focusing on the phenolic profile and AChE activity of extracts [19].

The aim of this study was to investigate the chemical composition of the extracts from bulb and aerial parts of *A. subhirsutum* by LC-MS^n^ analysis, and their antioxidant properties by DPPH, ABTS and TEAC assay were evaluated. In recent studies, garlic components proved to be active in neuroprotection and cancerogenesis due to the direct and indirect antioxidant properties [20]. Thus, in order to investigate the potential application of *A. subhirsutum* extracts in AD and cancer chemoprevention, the AChE inhibitory properties and cytotoxicity on tumor cells were also investigated.

## 2. Results

### 2.1. Chemical Composition of Extracts

**Figure 1:**
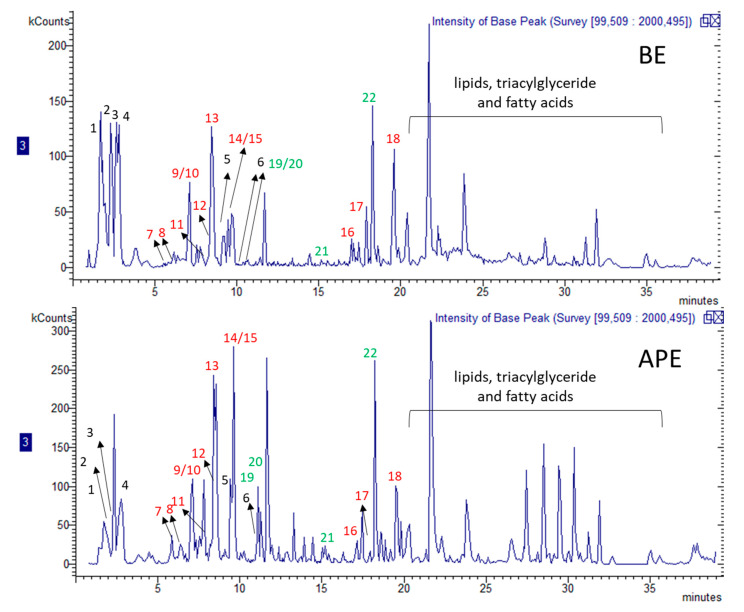
Chromatogram of BE and APE extract; number of compounds is related to Table 1: black = sulphur-containing compounds, red = flavonoid and phenol, green = amide phenylpropanoid derivatives.

The data obtained combining the DAD and MS^n^ data allowed to identify sulfur-containing compounds as well as flavonoids, polyphenols and tyramine ester with hydroxycinnamic acids. As reported in Figure 1 and Table 1, the same peaks were assigned to lipids, triacylglyceride and fatty acids. The extracts analyzed were obtained by a simple maceration in ethanol followed by concentration under vacuum. This extraction procedure allowed the extraction also of other constituents of plant material over sulfur-containing compounds. The identification of most common sulfur compounds was achieved using reference compound alliin and by comparison with garlic extract containing allicine, as well as the literature data. Regarding the typical fragments that are observed for alliin (retention time 9.44 min) from the molecular ion at *m/z* 178 and fragment at *m/z* 88, the same pattern is observed for a second peak (retention time 11.2 min) that was tentatively ascribed to cycloallin [21]. Other constituents identified are allicin, that present *m/z* 163 and fragments at *m/z* 73 and minor intense at *m/z* 87. Derivatives of aminoacids, in particular γ-glutamyl (S)-allylcysteine, γ-glutamyl-S-methylcysteine and γ-glutamyl-S-*trans*-propenyl cysteine, were identified on the basis of their characteristic fragment in MS^2^ at *m/z* 145.

Alliin and allicine were detected in both extracts. This was expected due to the fact that allicine is produced from alliin due to enzymatic activity of allynase. During plant drying and probably partly during extraction and concentration procedures this conversion may take place. In the extracts, allicine and alliin were present at 26.4 and 29.51 mg/g in BE, and 41.69 and 44.85 mg/g in APE being the most abundant sulfur compounds. The amount of cycloalliin, γ-glutamyl (S)-allylcysteine, γ-glutamyl-S-methylcysteine, and γ-glutamyl-S-*trans*-propenyl cysteine was lower compared to allicine and alliin, but measured in comparable amounts in BE and APE. γ-Glutamyl (S)-allylcysteine was detected in greater amount compared to other cysteine derivatives in both extracts. The content of all the identified sulfur compounds was 73.59 mg/g in BE and 104.86 mg/g in APE.

Literature data indicate that garlic contains on average 8 g/kg of allin, while in dried material the allin content is in the range of 20–25 mg/g. On the other hand, crushed raw garlic contains 37 mg/g of allicin [22]. A recently published paper reported, for fresh Croatian garlic, amounts ranging from 1–77 mg/g [11]. Thus, the allicin content in the Sicilian *A. subhirsutum* is consistent with that found in the Croatian samples.

Flavonoids and polyphenols were the most abundant class of constituents in the extracts, namely 118.77 mg/g in BE and 300.06 mg/g in APE. Analyses revealed significant contents of methoxy quercetin and luteolin aglycones, the most abundant ones in BE (22.19mg/g) and APE (53.26mg/g) respectively. LC-MS analysis identified five different quercetin derivatives, namely three methoxy quercetin isomers, tamarixetin (4′-O-methylquercetin) and 3,7-dimethylquercetin. Glucosyl gallate was also detected in both extracts in similar amounts.

Amide derivatives from hydroxycinnamic acids and tyramine were detected and the most abundant one was N-*trans*-feruloyl-tyramine. Such derivatives were previously reported also in *A. sativum* [23] and *A. fistolosum* L. [10]. Notably, when comparing the analyzed samples with the literature data reported for other garlic species [11], the hydroxycinnamic acid derivatives as ferulic acid and chlorogenic acid derivatives, were not detectable, thus suggesting a different polyphenol composition profile. These quali-quantitative differences can be related to species-specific phenolic compositions as well as due to the different area of cultivation and processing in the post-harvest procedures including drying and extraction protocols. The proposed method allows the quali-quantitative analysis of three different classes of Allium compounds, thus offering a valuable approach to assess differences of samples and study of phytochemical composition.

### 2.2. Antioxidant Activity

The antioxidant properties of *A. subhirsutum* extracts can be determined using different in vitro assays that are able to measure both radical scavenging capability and/or potential antioxidant. In our study, the antioxidant activity of ethanol extracts of the aerial parts and bulbs from *A. subhirsutum* was tested with the (DPPH•) and ABTS+ assays (Table 2), and the analysis showed a significant difference in the antioxidant capacity of the individual parts of garlic.

Data from DPPH assay showed that BE (IC_50_ = 1152 ± 3 μg/mL) had about two times lower antioxidant activity compared with APE (IC_50_ = 544 ± 1 μg/mL). The ability to reduce free radicals determined by ABTS, in different parts of garlic, varied. In APE (IC_50_ = 88.1 ± 7 μg/mL), the antioxidant capacity, as measured in the ABTS assay, was five times higher compared with BE (IC_50_ = 481 ± 26 μg/mL). In this assay, BE and APE showed a notable antioxidant activity, that was respectively 44 and 8 times lower than that of Trolox. In the DPPH assay, the two extracts showed a lower activity than that of Trolox, i.e., 73 and 34 times lower for BE and APE, respectively. The ability to reduce iron ions determined by the FRAP assay was similar for both extracts, (bulbs: TEAC = 263 ± 23.9 μmolTE/g; APE: TEAC = 357.9 ± 15.9 μmolTE/g).

Comparing the phytochemical contents, the amount of sulfur compound and polyphenols is 30% and 62% lower in BE compared to APE respectively. On the other hand, amide phenylpropanoid derivatives are nearly the same. As allicine and related compounds as well as flavonoids and polyphenols are well known for their antioxidant activities, the differences observed for the two extracts can be at least in part related to these differences in phytochemical composition.

### 2.3. AChE Inhibition

An important approach to treat Alzheimer’s disease (AD) is to enhance the acetylcholine level in the brain by inhibition of AChE and cholinesterase inhibitors from plants, which are considered as promising candidates [26]. Different studies have aimed to evaluate the effect of extracts [27] or essential oils from garlic bulbs on acetylcholinesterase (AChE) activity in vitro [28] and have revealed that garlic exerts an inhibition on this enzyme as well as possesses antioxidant properties. Allicine showed a potential to strongly inhibit AChE but weakly inhibit BuChE in a concentration-dependent manner. Allicine is able to upregulate the levels of acetylcholine in the brain [29], and these results provide an interesting basic contribution regarding the beneficial effects claimed for garlic that may be of therapeutic value in AD [29].

In our work, we observed a similar inhibitory activity for BE and APE and 1 g of extract corresponds to 6.7 and 8.6 mg of galanthamine, respectively (Table 3).

Thus, these extracts contain 3 to 4 mg of galanthamine equivalent for 500 mg, and this result could be considered significant for its in vitro effect. This activity can be suitable for a nutraceutical product considering that the doses of the alkaloid are in general in the range from 8 to 24 mg/die. A recently published paper reported the evaluation of AChE inhibitory properties of Turkey collected sample of *A. subhirtusum,* and evaluation of its phenolic constituents. We observed differences in absolute values measured for the plant extract with IC_50_ of 8,35 and 42,4 ug/mL for bulbs and aerial part, respectively. Also, the reference compound galanthamine presented in the paper an IC_50_ of 0.106 ug/mL. These differences can be ascribed to different experimental settings. We could compare the observed effects with relative potency of extracts compared with the reference compound. Inhibitory properties observed by Emir et al. indicate greater inhibitory properties for *A. subhirtusum* aerial parts compared to bulbs, while our data indicate similar activity for the two parts. Regarding the composition, authors reported for their sample, large amounts of hydroxybenzoic acid derivatives, ferulic and coumaric acid both in the bulbs and aerial parts with a difference in single compound amount [19]. No information is available about other classes of constituents. Our data showed differences in bulbs and aerial part inhibitory properties supported by significant differences in the amount of both phenolic and sulfur-containing compound as summarized in Table 1. The differences of the Turkey and Sicily samples may be ascribed to the variations in plant secondary metabolites due to climatic and geographic conditions.

Considering the quali-quantitative composition as indicated in Table 1 and the similar observed IC_50_ values for BE and APE, we could speculate that the inhibition of AChE could be ascribed to different classes of compounds. A previous study reported that allicin [29] showed a concentration-dependent inhibition of AChE and could have a potential to ameliorate the decline of cognitive function and memory loss associated with AD. Other studies indicated that also phenolic compounds such as quercetin possess significant AChE inhibitory properties, suggesting a role for inhibition of AChE in the analyzed extracts [30]. N-ferulyl tyramine demonstrated a moderate inhibition activity on AChE when tested as a pure compound [31], while extracts of *Salsola vermiculata* L., rich in N-feruloyl tyramine, exert significant AChE inhibition [32]. Overall, the bibliographic data indicate that AChE inhibitory activity may be related to the different classes of compounds detected in *A. subhirsutum*.

These preliminary data indicate the potential activity of *A. subhirsutum* extracts and open new questions related to the in vivo metabolic fate of allin and allicine, as well as of the other constituents, thus suggesting further studies in which the in vivo production of metabolites as well as the pharmacological activities of all the derivatives should be evaluated. Furthermore, organ distribution of these compounds after oral intake as well as in vivo behavioral test are needed to fully assess the possible role of garlic as a useful food agent in the management of AD or other metabolic and degenerative diseases.

### 2.4. Cytotoxicity on Tumor Cells

In order to evaluate other possible bioactivities of the *A. subhirsutum* extracts, the cytotoxic activity of BE and APE was evaluated considering three human tumor cell lines, i.e., HCT116 (colon carcinoma), MDA-MB231 (breast adenocarcinoma), and T98G (glioblastoma multiforme). In Table 4, the IC_50_ values of these extracts are reported.

Data analysis shows that extracts have a moderate antiproliferative effect on two of the three human tumor cell lines examined. In particular, HCT116 human carcinoma cell line appeared to be more susceptible to both extracts, whereas T98G human glioblastoma cell line resulted in being insensible, at least at the highest concentrations tested. A bit higher activity was shown for BE compared to APE in HCT116 and MDA-MB 231 cells.

There is traditional use of garlic both as food and medicine since ancient times in many different human populations. In ancient times, there are indications for the use of this remedy for intestinal disorders, respiratory infections, skin diseases, bacterial infections, worms, wounds, and tumors in Babylonians, Egyptians, Phoenicians, Greeks, and Romans. Many publications deal with allium species and their bioactivities, indicating significant interest by the scientific community on this natural product, especially in the area of cytotoxic and antitumor compounds. In this context, our data on *A. subhirsutum* represent new information showing moderate cytotoxic activity as previously reported [33,34,35,36,37].

Since the early studies of Weisberger and Pensky (1958) [38] that showed that thiosulfinate extracts from garlic were able to inhibit tumor cell grow both in vitro and in vivo, many other studies have been carried out. Most of the investigations considered the sulfur-containing compounds as the main active constituents considering them as chemopreventive or potential anticarcinogen agents [33].

The moderate cytotoxic activity could be attributed to some components present in these extracts. Several studies have reported that allicin can inhibit cell proliferation in a variety of tumor cells. It induces cytochrome c release from the mitochondria, activation of caspase-3, −8, −9, and increases Bax and Fas expression in the EL-4 lymphoma cells [39]. An in vitro study showed that allicin promoted the apoptosis and suppressed the survival and proliferation of HCT116 cells through the suppression of STAT3 signaling activation [40]. Quercetin inhibits growth of human SW480 colon cancer cells in association with inhibition of cyclin D1 and surviving expression through Wnt/beta-catenin signaling pathway [41]. Another study demonstrated the mechanism of quercetin-induced apoptosis via AMPK activation and p53-dependent apoptotic cell death in HT-29 cells and shows that the mechanism of cell cycle arrest is by AMPK phosphorylation. Moreover, quercetin induced apoptosis in HCT116 through Sestrin 2/AMPK/mTOR pathway, which was regulated by the increase of intracellular ROS [42]. Quercetin blocks the cell cycle at the G1/S phase in human colon cancer cells and in human leukemic T-cells, but in G2/M phase in human breast cancer cells [43]. It also mediates the downregulation of mutant p53 in the human breast cancer cell line MDA-MB468 and induces apoptosis in MDA-MB 231 [43]. Luteolin can suppress the proliferation of various kinds of tumor cells in vitro with IC_50_ from 3 to 50 μM, and inhibited tumor growth effectively in vivo when administered, e.g., in concentrations of 50 to 200 ppm in food, as indicated in a recent review [44]. The presence of allicine and different flavonoid derivatives in the extracts of of *A. subhirsutum* could justify the effects observed in colon carcinoma and human breast adenocarcinoma cell lines. Our results suggest the need for the evaluation of potential activity of fractions enriched in specific classes of compounds and purified constituents. This will allow to observe the polyphenols and sulfur-containing compounds’ activity separately, as well as allowing to compare the cytotoxicity of fractions and purified compounds to observe potential synergistic effects. Finally, the use of different cell lines will be also considered in order to evaluate potential specificity of constituents or fractions.

The antioxidant activity of *Allium* spp. has been ascribed to phenolic derivatives and sulphur-containing compounds [33,45]. The obtained data on the antioxidant tests are in agreement with the quantitative measurements of phytoconstituents. In fact, the APE extract showed higher levels of sulfur-containing compounds, polyphenols, while feruloylamides were contained in similar amounts in both extracts.

In this context, the well-documented antioxidant activity for allicine derivatives suggest that this class of constituents can be ascribed as the main reason responsible for the antioxidative properties of this species [46]. On the other hand, some authors suggest that other bioactive compounds such as dietary fibers, microelements (especially Se) and polyphenols [47] could contribute to these effects. Related to the possible health-promoting activities of garlic extract, literature data indicate that allicin can prevent the lipid peroxidation of liver homogenate in a concentration-dependent manner [48]. The results suggest that allicin lacks direct hydroxyl radical scavenging activity but shows antioxidant activity, probably mediated via its ability to inhibit enzymes that promote pro-oxidant status via thiol exchange [49]. Moreover, according to scientific literature [50], a moderate positive correlation was found between the total phenolic content and the antioxidant activity of garlic [51]. In any case, due to the high instability of allicin and due to the easy opportunity for this compound to be converted in many other derivatives, the overall evaluation of the presence of different compounds in each tested extract can be important for understanding what compounds are responsible for the observed effects. For this reason, an accurate phytochemical profile was achieved in order to assess the quali-quantitative composition of different plant extracts. Our phytochemical investigations (Table 1) showed that APE was richer in polyphenols, in particular methoxy quercetin, quercetin and luteolin, which are considered valuable antioxidants [52,53,54]. On the other hand, the amount of phenolic compounds in BE is nearly one third compared to APE. These data justify the differences observed for the antioxidant activity. Our study also confirms the results obtained in previous researches; in fact, these studies showed that extracts from the leaves and flowers of wild-type species of Allium are more active and efficient in respect to bulbs. In these studies, differences were observed also in aged extracts of leaves and bulbs (tested up to 20 months) of *Allium* (*A. neapolitanum* Cyr., *A. roseum* L., *A. subhirsutum*) [55], which reported that aged extracts obtained from leaves showed the best antioxidant activity, followed by those of flowers and bulbs. Four amide phenylpropanoid derivatives were identified in the extract, and the most abundant was the N-feruloyl tyramine. Previous studies investigated its antioxidant activity by the DPPH assay and compared its activity with that of other feruloyl derivates. A good activity, slightly below that of quercetin, was observed by [31]. Thus, the anti-scavenger properties observed may also be related to this class of phytoconstituents, also considering the similar amount of phenylpropanoid amides detected in BE and APE.

## 3. Materials and Methods

### 3.1. Plant Material

Aerial parts (flower and stems) and bulbs of *Allium subhirsutum* were collected 2 km west of Madonnuzza, Petralia Soprana, Palermo (Sicily, Italy) (37°47′44” N, 14°06′47” E, 1020 m a.s.l.), at the end of May 2018, from plants at the full flowering stage. Typical specimens (PAL 18/85), identified by Mr. Emanuele Schimmenti, have been deposited in the Department STEBICEF, University of Palermo, Palermo, Italy.

### 3.2. Plant Extraction

The dried aerial parts and bulbs were pulverized by using an electric blender. About 100 g of both samples were soaked in absolute ethanol (≥99.8%) in 1000 mL volumetric flasks. The flasks were covered and left for 48 h. The content of the flasks was then filtered, and the filtrates were concentrated by using a rotary evaporator to give solid residues (yield: 5.38% for aerial parts (APE) and 2.68% for bulbs (BE), respectively). The dried residues were stored in an air-sealed analytical container.

### 3.3. HPLC-ESI-MS^n^ Analysis

The analysis of *A. subhirsutum* constituents was obtained using an LC-ESI-MS^n^ approach. An Agilent 1260 (Santa Clara, CA, USA) chromatograph equipped with a diode array detector (DAD) and Ion Trap (IT) mass spectrometer Varian MS-500 (Varian inc. Palo Alto, CA, USA) As the stationary phase, an Agilent Eclipse XDB C-18 (3.0 × 150 mm, particle size 3.5 μm) was used. Binary eluent system formed by 0.1% formic acid in water (A) and acetonitrile (B) was applied. The mobile phase composition began with 90% A and changed to 0% of A in 33 min; the flow rate was 0.4 mL/min and the sample injection volume was 10 µL. To quantify the main constituents, we used some reference compounds that are commercially available, namely rutin (Sigma Aldrich, St. Louis, MO, USA) and S-Allyl-L-cysteine sulfoxide –Alliin- (Sigma Aldrich, Milano, Italy). Allicine content was estimated using alliin calibration curve, while rutin was used to quantify the flavonoid derivatives. On the basis of UV spectra observed for the peaks, the wavelength of λ 240 nm was selected for alliin and allicine; while the λ of 350 nm was adopted for flavonol derivatives. For the preliminary identification of the classes of compounds, the UV-Vis spectra were observed and acquired in the range of 200–400 nm. To tentatively establish the structure of the eluted compounds, MS spectra were acquired in the *m/z* 50–2000 range, using ESI ion source operating in positive ion mode; to assess the fragmentation pattern of detected constituents, the turbo data-dependent scanning (tdds^®^) option of the instrument was used. The parameters of negative and positive ion modes applied for each sample were as follows: drying temperature, 285 °C; needle voltage, 5 kV; drying gas pressure (nitrogen), 25 psi; and nebulizer gas pressure (air), 35 psi. Data were recorded within a mass scan mode of 15,000/s. The mass scan average was set at three microscans (2.19 s/scan). For quantification of the identified compound was use as standard allicin for sulfur-containing compound, and rutin for flavonoid and calibration curves were obtained preparing fresh solution from 50–5 ug/mL and 85–8.5 ug/mL, respectively.

### 3.4. Antioxidant Assays

The use of multiple radical generating systems provides a better insight into the measure of antioxidant potential power, and DPPH, ABTS and FRAP assay were used to evaluate the scavenging activity and ferric-reducing ability of different *A. subhirsutum* extracts. All assays were conducted in a 96-well microplate assay following previously-described protocols [56]. For all analyses, a standard curve was prepared using different concentrations of Trolox, and all determinations were performed in triplicate. The antiradical activity was expressed as IC_50_ (μg/mL), which represented the extract concentrations scavenging 50% of DPPH or ABTS+ radicals or as Trolox equivalent antioxidant capacity (TEAC).

### 3.5. AChE Inhibitory Assay

Activity of AChE in different extracts was determined with an adapted microplate assay according to the method of Ellman et al., [57] using Electric eel acetylcholinesterase, and acetyl thiocholine iodide (ATCI) as a substrate of the reaction as previously described [58].

### 3.6. Cell Culture and Sample Treatment

HCT116 human colon carcinoma, MDA-MB 231 breast adenocarcinoma, and T98G glioblastoma multiforme cell lines were provided by the American Type Culture Collection (ATCC, Manassas, VA, USA). The HCT116 cells were maintained in Roswell Park Memorial Institute medium (RPMI-1640; Corning, Manassas, VA, USA) supplemented with 100 IU/mL penicillin, 100 μg/mL streptomycin (Corning, Manassas, VA, USA), 2 mM L-glutamine (Corning, Manassas, VA, USA), and 10% of heat inactivated bovine fetal serum (FBS-HI) (Corning, Manassas, VA, USA). MDA-MB 231 cell line was maintained in medium Dulbecco (DMEM; Corning, Manassas, VA, USA) supplemented with 100 IU/mL penicillin, 100 μg/mL streptomycin, 2 mM L-glutamine, and 10% of FBS-HI. The T98G cell line was maintained in Minimum Essential Medium (MEM; Corning, Manassas, VA, USA) with 2mM of L-glutamine, 0.1 mM of non-essential amino acids (PAA Laboratories; GE Healthcare Life Sciences, Chalfont, UK), 1 mM of sodium pyruvate (PAA Laboratories; GE Healthcare Life Sciences, Chalfont, UK), 100 IU/mL of penicillin G, 100 μg/mL streptomycin, and 10% of FBS-HI. All cell lines were kept in an incubator at 37 °C, in a humidified atmosphere with 5% CO_2_. Cells were maintained in culture by detachment with Tripsin/EDTA and diluted in fresh medium before reaching the cell confluence state (approximately 80% confluence). Exponentially growing cells were plated at 2 × 10^4^ cells/mL into 96-well microtiter tissue culture plates (Corning Incorporated, NY, USA) and incubated for 24 h before the addition of *A. subhirsutum* extracts.

### 3.7. Cytotoxicity Assay

The reduction of tetrazolium salt, MTT (Sigma-Aldrich, Milano, Italy), to formazan by mitochondrial succinate dehydrogenase was used to evaluate the viability of the tumor cells as previously described [59]. After 24 h from seeding the cells in culture plates, the *A. subhirsutum* extracts were added to the medium in a concentration range of 0.78 to 200 μg/mL. Cisplatin was used as a positive control in a concentration range of 0.05 to 50 µg/mL. After 72 h of incubation, 10 μL of the MTT (5 mg/mL in PBS solution) was added to each well and the plates were incubated at 37 °C for a further 4 h in a humidified atmosphere with 5% CO_2_. The intracellular reduction of tetrazolium salts by the enzyme succinate dehydrogenase causes the formation of blue formazan crystals at the bottom of the well. At the end of the incubation time, the supernatant was removed and 100 μL of dimethyl sulfoxide (DMSO; Sigma-Aldrich, Milano, Italy) was added per well. The plates were stirred for about 15 min in order to solubilize the crystals formed and allowed to read the absorbance at a wavelength of 540 nm with the OMEGA plate reader from BMG Labtech (Durham, NC, USA). Cell viability was calculated as a percentage ratio of the absorbance of the sample to the control. Every concentration was repeated in three wells.

## 4. Conclusions

The present study assessed the chemical profile, the antioxidant properties, the AChE inhibition, and cytotoxic activity of bulbs and aerial parts of *A. subhirsutum* (“aglio pelosetto”) growing in Sicily (Italy); three different classes of phytoconstituents were analysed. Alliin and allicine, quercetin derivatives, and luteolin were identified as the most abundant compounds in both parts. Furthermore, amides of tyramine were also detected. The identified compounds were present in 73 and 104 mg/g for BE and APE, which is in the range of what is reported in the literature for other garlic species. In this regard, our measurements showed a significant amount of alliin present in the samples, thus indicating the abundance of sulphur compounds in this species. Phenolics were revealed in an amount of 119 and 300 mg/g for BE and APE, while amide phenylpropanoid derivatives revealed in both extracts are in a similar range of 50 mg/g. Regarding the bioassays, significant antioxidant activity was observed with aerial parts being more active than bulbs. In agreement with the antioxidant results, the phytochemical analysis revealed that the more active extract, APE, contains a higher amount of sulfur-containing compound and phenolic compared to BE. Both extracts exhibited a similar degree of AChE-inhibitory properties, while limited cytotoxic activity was observed towards the considered human tumor cell lines. Since the selected extracts have shown inhibitory properties on AChE and present antioxidant activity, these results may provide an initial contribution to the possible development of a nutraceutical-targeting degenerative disease and presenting beneficial effects on health. This work, starting from the nutritional use of *A. subhirsutum*, and due to the claimed effects on health related to garlic, underline the potential usefulness of these extracts and suggest the need for further studies to assess its potential use as an adjuvant in AD treatment.

## Figures and Tables

**Table 1:** Compounds identified in extracts from bulb (BE) and aerial parts (APE) of *A. subhirsutum* by LC-MS. rt: retention time, nd: not detected, * Food Database (https://foodb.ca/): A = FDB000604, B = FDB018376, C = FDB000176, D = FDB016383 E = FDB001548.

n	rt (min)	Compound	[M + H]^+^	Fragments	BE (mg/g)	APE (mg/g)	Ref
1	1.64	Allicine	163	87 73	26.40 ± 0.02	41.69 ± 0.02	[21]
2	1.89	Gamma-glutamyl (S)-allylcysteine	291	145	10.97 ± 0.02	14.55 ± 0.02	[21]
3	2.85	Gamma-glutamyl-S-methylcysteine	265	145 136 139	1.21 ± 0.01	1.28 ± 0.01	[21]
4	3.00	Gamma-glutamyl-S-trans-propenyl cysteine	291	145	4.00 ± 0.02	0.59 ± 0.01	[21]
5	9.44	Alliin	178	88	29.51 ± 0.04	44.85 ± 0.04	[21]
6	11.2	Cycloalliin	178	88	1.51 ± 0.01	2.04 ± 0.01	[21]
		**sulfur compounds**		**tot**	**73.59**	**104.86**	
7	6.91	Methoxy quercetin trisaccharide isomer 1	779	493 317 302 284 257	0.64 ± 0.01	0.96 ± 0.04	[24]
8	7.13	Methoxy quercetin isomer 1	317	302 284 257	16.57 ± 0.02	20.04 ± 0.01	*A
9	7.76	Quercetin	303	285 257 229 166	4.28 ± 0.02	23.5 ± 0.02	[25]
10	7.76	Methoxy quercetin trisaccharide isomer 2	779	493 331 317 302 284 257	3.84 ± 0.02	9.91 ± 0.02	[24]
11	8.45	Luteolin	287	213 166	20.19 ± 0.03	53.26 ± 0.02	[25]
12	8.55	Methoxy quercetin isomer 2	317	302 284 257	22.19 ± 0.02	28.47 ± 0.02	*A
13	8.86	Glucosyl gallate	333	315 297 268	21.61 ± 0.04	26.13 ± 0.03	*B
14	9.45	kaempferol	287	259 241 213	6.79 ± 0.01	26.86 ± 0.03	[25]
15	9.67	Methoxy quercetin isomer 3	317	302 284 257	9.17 ± 0.05	48.18 ± 0.10	*A
16	17.4	3,7-Dimethylquercetin	331	314 296 265	10.70 ± 0.04	26.37 ± 0.03	*C
17	17.99	Tamarixetin (3,3′,5,7-Tetrahydroxy-4′-methoxyflavanone)	317	301 271 264 255 239 212	2.77 ± 0.01	9.78 ± 0.02	*D
18	19.58	5,3′,4′-Trihydroxy-3-methoxy-6,7-methylenedioxyflavone	345	329 297	nd	26.60 ± 0.05	*E
		**flavonoid and polyphenol**		**tot**	**118.77**	**300.06**	
19	11.4	coumaroyl-tyramine	284	149 147 121 120 91	1.20 ± 0.01	1.34 ± 0.01	[10]
20	11.7	N-trans-Feruloyl-tyramine	314	178 149 147	41.88 ± 0.10	43.89 ± 0.03	[10]
21	15.3	Coumaroyl-octopamine	300	178	nd	0.78 ± 0.01	[23]
22	18.55	N-trans-Feruloyl-3-methoxytyramine	344	178 149 147	3.64 ± 0.02	6.62 ± 0.02	[10]
		**amide phenylpropanoid derivatives**		**tot**	**46.71**	**52.62**	

**Table 2:** Antioxidant activity of the ethanolic extracts from bulb (BE) and aerial parts (APE) of *A. subhirsutum.*

Extract	DPPH		ABTS		FRAP
	TEAC ^a^ μmolTE/gr	IC_50_ μg/mL	TEAC μmol TE/gr	IC_50_ μg/mL	TEAC μmol TE/gr
BE	54.44 ± 1.2	1152 ± 3	90.53 ± 0.9	481 ± 26	263 ± 23.9
APE	115.3 ± 2.7	544 ± 1	512.42 ± 4.9	88.1 ± 7	357.9 ± 15.9
Trolox		15.7 ± 0.4		10.9 ± 0.6	

^a^ TEAC = Trolox equivalent (TE) antioxidant concentration.

**Table 3:** AChE inhibitory properties of the ethanolic extract from bulb (BE) and aerial parts (APE) of *A. subhirsutum*.

	IC_50_ μg/mL	mgGEIC/g ^a^
BE	675 ± 14	6.66 ± 0.15
BEAPE	526 ± 5	8.55 ± 0.29
**Galantamine**	4.5 ± 0.2	

^a^ GEIC = galantamine-equivalent inhibition capacity.

**Table 4:** Cytotoxicity of the ethanolic extract from bulb (BE) and aerial parts (APE) of *Allium subhirsutum*, expressed as IC_50_ values on human tumor cell lines.

Extracts	Cell line (IC_50_ µg/mL) ^a^		
	HCT116 ^b^	MDA-MB 231 ^c^	T98G ^d^
**BE**	71.07	73.72	>200
95% C.I. ^e^	62.71–80.54	65.37–83.14	
**APE**	99.17	140.4	>200
95% C.I. ^e^	91.25–107.8	135.6–145.3	
**Positive control**			
Cisplatin	2.62	2.07	2.34
95% C.I. ^e^	2.41–2.85	1.56–2.22	2.05–2.56

^a^ IC_50_ = The concentration of compound that affords a 50% reduction in cell growth (after 72 h of incubation). ^b^ Human colon carcinoma cell line. ^c^ Human breast adenocarcinoma cell line. ^d^ Human glioblastoma multiforme cell line. ^e^ Confidence interval.
